# Host DDX Helicases as Possible SARS-CoV-2 Proviral Factors: A Structural Overview of Their Hijacking Through Multiple Viral Proteins

**DOI:** 10.3389/fchem.2020.602162

**Published:** 2020-12-10

**Authors:** Flavia Squeglia, Maria Romano, Alessia Ruggiero, Giovanni Maga, Rita Berisio

**Affiliations:** ^1^Institute of Biostructures and Bioimaging (IBB-CNR), Naples, Italy; ^2^Institute of Molecular Genetics (IGM-CNR), Pavia, Italy

**Keywords:** SARS-CoV-2, COVID19, protein structure, viral infection, DDX helicases

## Abstract

As intracellular parasites, viruses hijack the host cell metabolic machinery for their replication. Among other cellular proteins, the DEAD-box (DDX) RNA helicases have been shown to be hijacked by coronaviruses and to participate in essential DDX-mediated viral replication steps. Human DDX RNA helicases play essential roles in a broad array of biological processes and serve multiple roles at the virus-host interface. The viral proteins responsible for DDX interactions are highly conserved among coronaviruses, suggesting that they might also play conserved functions in the SARS-CoV-2 replication cycle. In this review, we provide an update of the structural and functional data of DDX as possible key factors involved in SARS-CoV-2 hijacking mechanisms. We also attempt to fill the existing gaps in the available structural information through homology modeling. Based on this information, we propose possible paths exploited by the virus to replicate more efficiently by taking advantage of host DDX proteins. As a general rule, sequestration of DDX helicases by SARS-CoV-2 is expected to play a pro-viral role in two ways: by enhancing key steps of the virus life cycle and, at the same time, by suppressing the host innate immune response.

## Introduction

COVID-19 is a respiratory disease caused by a novel enveloped, positive-sense, single-stranded RNA betacoronavirus, designated as SARS-CoV-2. Mechanistically, SARS-CoV-2 enters the cell through the binding of the spike protein to the ACE2 receptors, as previously observed for SARS-CoV (Luan et al., 2020; Romano et al., 2020a; Wrapp et al., 2020). Then, the human transmembrane protease serine 2 (TMPRSS2) hydrolyses and activates the spike protein (Hoffmann et al., 2020). An additional protease, possibly furin, is also involved in this process (Lukassen et al., 2020). Spike proteolysis allows SARS-CoV-2 to enter the cells by endocytosis or by direct fusion of viral and host membranes (Xia et al., 2020; Yang and Shen, 2020). The infecting RNA produces messenger RNA (mRNA), which will be then translated by host ribosomes into protein products (Walsh et al., 2013; Romano et al., 2020b). Using the genetic information encoded in mRNA, the virus takes advantage of the host cell to produce all the components needed for the generation of new viral particles.

Host-pathogen interactions form the basis of the pathogenicity of viruses, including highly pathogenic emerging viruses such as Ebola, SARS-CoV, MERS-CoV, and SARS-CoV-2. By analogy with other known coronaviruses, the RNA replication machinery of SARS-CoV-2 is expected to be regulated by a diversity of host factors, including cellular RNA helicases involved in key events of viral infection (van Hemert et al., 2008; Ranji and Boris-Lawrie, 2010; Sharma and Boris-Lawrie, 2012). Among those, DEAD-box (DDX) RNA helicases are emerging as key players in the host-pathogen interaction network, by modulating innate immunity and viral proliferation in multiple ways. DDX helicases are involved in many steps of the RNA metabolism, including RNA-RNA and RNA-protein remodeling in an ATP-dependent manner (Hilbert et al., 2009; Linder and Jankowsky, 2011). Given the essential role of DDX proteins in many biological processes in humans, their mutation or mis-regulation correlate with an increasing number of pathological processes, including oncogenesis, inflammation, viral replication, and immune response (Steimer and Klostermeier, 2012).

In host-pathogen interactions, DEAD-box helicases display diverse functions upon viral invasion, where they can act as positive or negative regulators of viral replication at different levels (Taschuk and Cherry, 2020). Specifically, DDX enzymes have shown to promote interferon (IFN) induction or other inflammatory signaling, leading to antiviral immunity (Soulat et al., 2008). Some of them function as cytoplasmic sensors of viral RNA, such as the canonical DDX58/RIG-1, while some others act in concert with other proteins (Zhang et al., 2011; Yoo et al., 2014). DDX proteins are required for replication of a number of human viral pathogens, such as HIV-1, HCV, Influenza A, Dengue, Infectious Bronchitis Virus (IBV)-CoV, and SARS-CoV (Fang et al., 2004; Goh et al., 2004; Chen J. Y. et al., 2009; Xu et al., 2010; Diot et al., 2016). Among these, HIV-1 is the most well-described case where the virus hijacks host helicases, such as DDX1, DDX3X, DDX5, and DDX17, to support reverse transcription, transcription, and nuclear export (Yasuda-Inoue et al., 2013).

**Figure 1:**
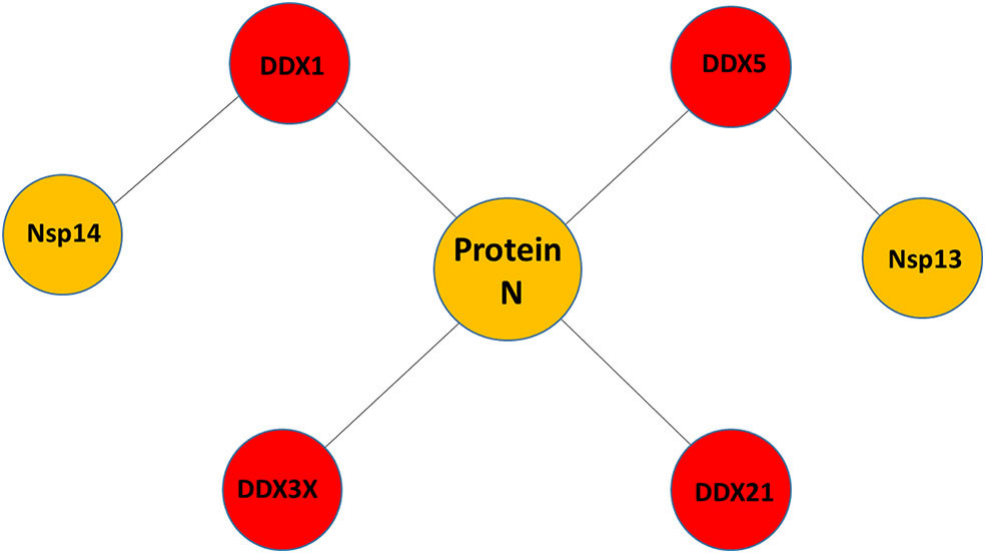
Pattern of putative interactions between host DDX helicases and SARS-CoV-2 proteins. These interactions were experimentally observed in other coronaviruses sharing highly conserved viral proteins with SARS-CoV-2.

Little is known about the potential role of DDX helicases in SARS-CoV-2 replication. Although coronaviruses carry their own RNA helicases, they hijack DDX proteins to positively modulate genome transcription and virus proliferation, acting as pro-viral effectors (Chen J. Y. et al., 2009; Xu et al., 2010). Specifically, SARS-CoV has been shown to recruit host helicases, such as DDX1 and DDX5, a feature observed in other coronaviruses, such as IBV-CoV (Chen Y. et al., 2009; Xu et al., 2010). A number of RNA viruses hijack DDX5 and DDX3X helicases, thus facilitating various steps of their replication cycles, although details of interactions and on the interference of host-pathogen interaction on the DDX functional states are lacking (Cheng et al., 2018). Gathering functional data from several coronaviruses, it is clear that the nucleocapsid (N) protein, a crucial protein in the coronaviral life cycle, plays a central role in the hijacking mechanism, although other molecular actors, like Nsp13 and Nsp14, have been identified in SARS-CoV (Figure 1, Table 1). The high sequence identities of the molecular players involved in these interactions suggests that viral replication of SARS-CoV-2 can be modulated by the host cell machinery in a similar manner. This review summarizes structural information on the host-pathogen interaction processes mediated by DDX helicases in coronaviruses, with an emphasis on SARS-CoV-2. In fact, since the molecular processes involved in these events are highly conserved among coronaviruses, it is likely that similar mechanisms operate in the replication of the novel SARS-CoV-2.

**Table 1:** Viral proteins known to interact with host DDX helicases in CoVs.

**Viral protein**	**CoV**	**Host DDX helicase**	**References**	**Hypothetic advantages of protein-mediated DDX hijacking in SARS-CoV-2**
Protein N	IBV-CoV	DDX3X	Emmott et al., 2013	Inhibition of DDX3X-mediated antiviral immune response
	IBV-CoV SARS-CoV-2	DDX1/DDX21 DDX21	Emmott et al., 2013; Gordon et al., 2020	Evasion of innate immune response through the inhibition of the viral sensor DDX1-DDX21-DHX36
Nsp14	IBV-CoV, SARS-CoV	DDX1	Xu et al., 2010	DDX1-mediated enhancement of the catalytic activity of Nsp14
Nsp13	SARS-CoV	DDX5	Chen J. Y. et al., 2009	Enhancement of viral RNA transcription and repression innate immunity

## A Structural Overview of SARS-CoV-2 Proteins Likely Involved in DDX Hijacking

### Nucleocapsid (N) Protein, a Crucial Protein in Coronaviral Life Cycle

Like the other coronaviruses, SARS-CoV-2 has four major structural proteins: the spike (S), the membrane (M), the envelope (E), and the nucleocapsid (N) protein. While the S, M, and E proteins make up the virion envelope, the N protein is located inside the virus particle where it binds with viral RNA (Siu et al., 2008). The N protein plays a primary role in protecting genomic RNA by forming the ribonucleoprotein (RNP) complex, which subsequently condensates through the interaction with the M protein (Narayanan et al., 2000).

In addition to its role in RNP formation, the N protein is a multifunctional phosphoprotein with pivotal roles in several events of the viral life cycle, such as regulating viral RNA synthesis (Chang et al., 2014; McBride et al., 2014; Cong et al., 2020). In fact, a number of studies showed that the N protein co-localizes with components of replication-transcription complex (RTC) at the early stage of CoV infection. Also, it interacts with the transmembrane protein Nsp3 of SARS-CoV to stimulate the infectivity of genomic RNA (Hurst et al., 2010). The N protein is post-translationally phosphorylated, a process that allows discrimination in the binding viral vs. non-viral mRNA, suggesting a pleotropic effect in RNA regulation (Spencer et al., 2008). So far, multiple investigations underlined the regulatory role of the N protein in viral replication or transcription, with the common view that the N protein has RNA-binding and chaperone activities (Cong et al., 2020). The N protein is abundantly expressed during infection and is highly immunogenic, capable of inducing a protective immune response against CoV (He Y. et al., 2004). Furthermore, it is also involved in the modulation of the host cellular machinery, by perturbing cellular events such as gene transcription, interferon production, actin reorganization, host cell cycle progression, and apoptosis (McBride et al., 2014). Consistently, the N protein of CoV has been reported to interact with numerous host cell proteins, including hnRNP-A1 (Wang and Zhang, 1999), B23 phosphoprotein (Zeng et al., 2008), Smad3 (Zhao et al., 2008), chemokine Cxcl16 (Zhang et al., 2010), translation elongation factor-1 alpha (Zhou et al., 2008), pyruvate kinase (Wei et al., 2012), and 14-3-3 (Surjit et al., 2005).

**Figure 2:**
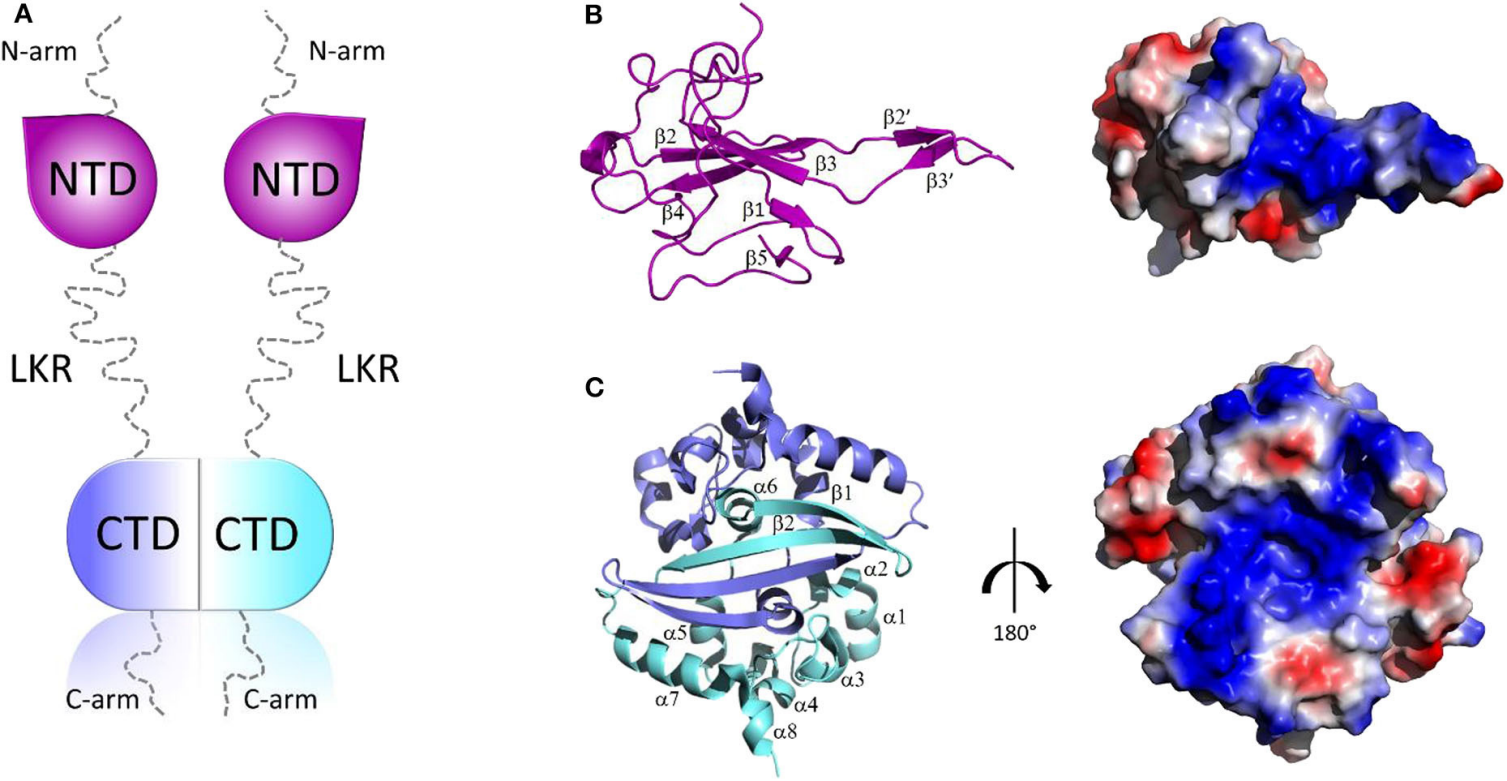
Structural overview of SARS-CoV-2 N protein. **(A)** Schematic representation of SARS-CoV-2 N protein; **(B,C)** Cartoon representations and corresponding electrostatic potential surface charge distributions of SARS-CoV-2 NTD **(**PDB code: 6M3M) and SARS-CoV-2 CTD (PDB code:6YUN), respectively.

Structurally, all CoV N proteins share the same modular organization (Jayaram et al., 2006; Saikatendu et al., 2007), consisting of two structural and independently folded domains, noted as the N-terminal domain (NTD) and C-terminal domain(CTD), flanked by three intrinsically disordered regions (IDR): an N-terminal arm (N-arm), a central linker region (LKR), and a C-terminal tail (C-tail) (Figure 2A). Although the full-length structure at atomic resolution is lacking, 3D structural information of the structured domains, together with biochemical data, provide crucial insights into the RNP formation process as well as other regulatory functions. Several studies have shown that both the NTD and CTD domains are responsible for the binding of viral RNA genome, whereas CTD also contributes to N protein oligomerisation (Huang et al., 2004; Chang et al., 2005, 2013; Yu et al., 2005; Chen et al., 2007; Chen I. J. et al., 2013; Lo et al., 2013). Interestingly, the LKR linker can modulate the process of viral genome packing, allowing the two structured domains to adopt a wide range of conformations (Chang et al., 2009) and is crucial for N protein oligomerisation (He R. et al., 2004; Luo et al., 2006). The LKR includes a Ser/Arg-rich (SR rich) region that contains a number of putative phosphorylation sites, which regulates N protein function during the early replication step of the viral RNA synthesis (Surjit et al., 2005; Lin et al., 2007; Peng et al., 2008; Wu et al., 2009). The kinase responsible for the phosphorylation of this SR-rich motif is the glycogen synthase kinase-3 (GSK-3), which is conserved in both JHMV and SARS-CoV (Wu et al., 2014). Consistently, treatment with GSK-3 inhibitor reduces N protein phosphorylation and the viral titer and cytopathic effects (Wu et al., 2014).

The 3D structures of the NTD and the CTD of SARS-CoV-2 were recently released (PDB codes: 6M3M and 6YUN, respectively), revealing high similarity with those of other CoVs (Ye et al., 2020). The NTD domain adopts a U-shaped β-platform structure containing a five stranded antiparallel β-sheet with the topology β4-β2-β3-β1-β5 (Figure 2B), with the two strands β2′ and β3′ located on a protruding β-hairpin (Figure 2B). This fold creates a positively charged pocket between which represents the RNA binding site, as confirmed by NMR studies on SARS-CoV NTD (Chang et al., 2014). Different features characterize the CTD domain, which forms a tightly intertwined homodimer (Figure 2C). Each CTD monomer presents an α-β fold with α1-α2-α3-α4-α5-α6-β1-β2-α7-α8 topology and the β1-β2 hairpin of the two monomers form an antiparallel β-sheet at the dimer interface (Yu et al., 2005; Lo et al., 2013), stabilized by extensive hydrogen bonding (Figure 2C). The dimer is also strongly stabilized by hydrophobic interactions between the helix α7 of one monomer and the β-sheet of an adjacent monomer and between helices α5 and α6 of the two monomers (Figure 2C). Solution NMR studies have confirmed that CTD exists predominantly as a dimer in the absence of nucleic acids (Chen et al., 2007). Nevertheless, X-ray crystallography and biochemical studies have shown that CTD is able to form further transient self-associations, conferring to the N protein the ability to form high-order oligomers (Chen et al., 2007; Chang et al., 2013). It has been hypothesized that N protein oligomerisation allows the optimal packaging of the RNA genome during RNP formation process (Chen et al., 2007; Takeda et al., 2008; Chang et al., 2009, 2014). As in the case with NTD, RNA-binding sites on the CTD have strongly positive electrostatic potential surfaces (Figures 2B,C) (Huang et al., 2004; Takeda et al., 2008; Chang et al., 2014), indicating non-specific electrostatic interactions between the N protein and the viral RNA (Chang et al., 2014), as expected for a protein that has to bind to diverse RNA sequences with reasonable affinity during encapsidation.

### Nsp13 Helicase, a Multifunctional Enzyme Involved in Genome Unwinding and the First Step in mRNA Capping

Helicases are ubiquitous motor proteins, also present in (+) RNA viruses with genomes larger than 7 kb. They are nucleic acid–dependent ATPases capable of unwinding DNA or RNA duplex substrates during nucleic acid replication, transcription, DNA repair, RNA maturation, and splicing (Patel and Donmez, 2006). Viral helicases belong to three out of the six currently recognized super-families: SF1, SF2, and SF3 (Lehmann et al., 2015). Sequence analysis suggests that Nsp13 of SARS-CoV-2 is part of the SF1 superfamily and can exert multiple enzymatic activities. Consistently, biochemical studies have shown that SARS-CoV Nsp13 unwinds both RNA and DNA duplexes in the 5′ to 3′ direction and is able to hydrolyze deoxyribonucleotide and ribonucleotide triphosphates (Ivanov and Ziebuhr, 2004; Adedeji et al., 2012). This reaction characterizes the first step of an important process of viral RNA synthesis, the protection of nascent mRNAs at their 5′ ends by a cap structure, which makes viral mRNA more stable and able to evade the host immune response. Therefore, not only is Nsp13 involved in genome unwinding, but, due to its RNA 5′-triphosphatase activity, it is an essential enzyme in the mRNA capping (Ivanov and Ziebuhr, 2004; Adedeji et al., 2012).

**Figure 3:**
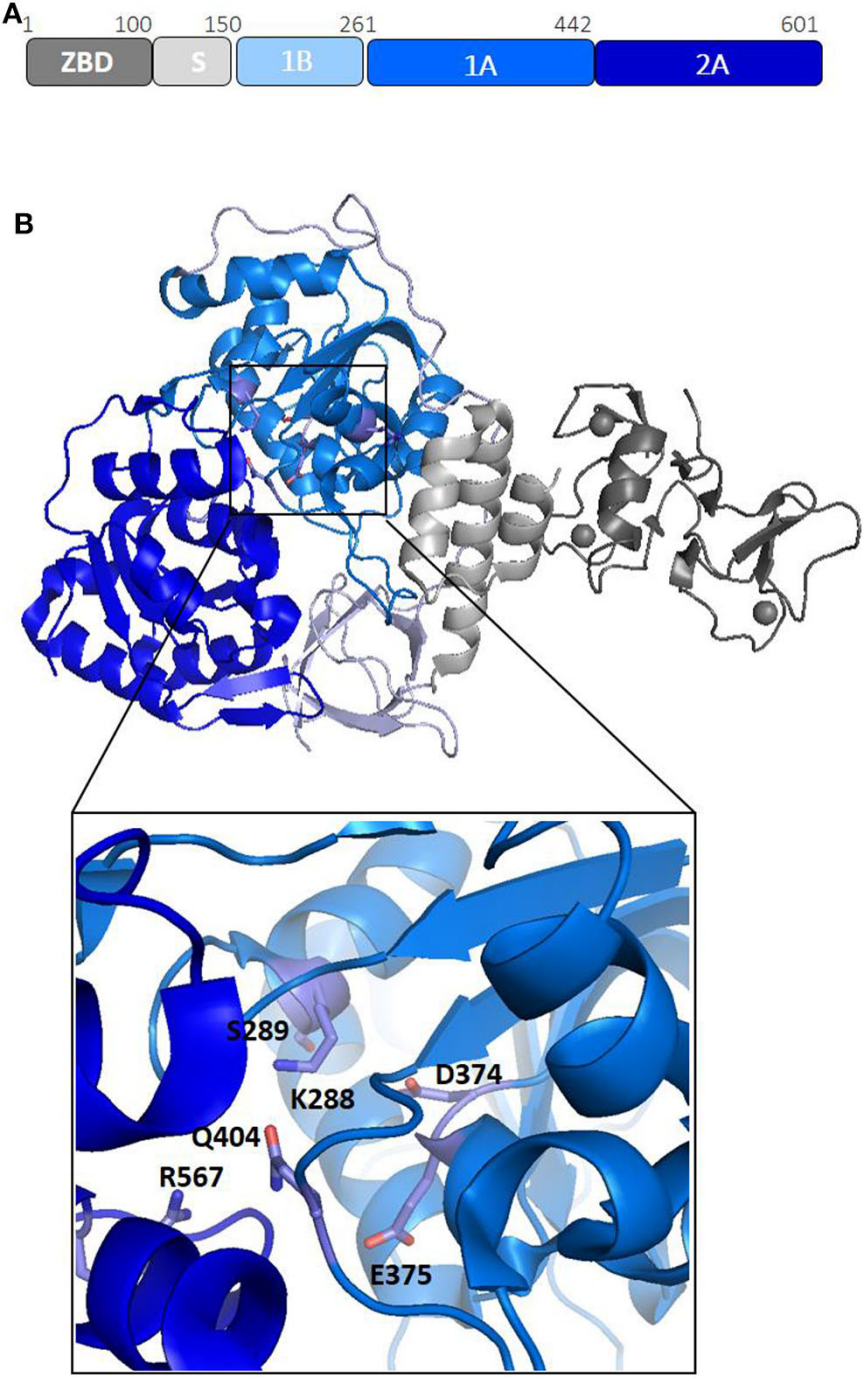
Structural organization of SARS-CoV-2 Nsp13 helicase. **(A)** Organization of Nsp13 in domains. **(B)** Cartoon representation of the crystal structure of Nsp13 from SARS-CoV-2 Nsp13 (pdb code 6zsl). The color code, in panel A, is: ZBD-gray, stalk-light gray, 1B-light blue, 1A-blu marine, and 2A-blue. In the inset, the key conserved residues responsible for NTP hydrolysis are drawn as sticks.

Nsp13 is conserved in all coronaviruses and is key to viral replication (van Dinten and van Tol H, 2000; Lehmann et al., 2015). Given the high sequence conservation and indispensability across all CoV species (Lehmann et al., 2015), Nsp13 is a promising target for the development of anti-viral drugs (Shum and Tanner, 2008; Adedeji et al., 2014). Structures of Nsp13 helicases from coronaviruses SARS-CoV and MERS and, more recently, SARS-CoV-2, have been hitherto reported (Hao et al., 2017; Jia et al., 2019). The Nsp13 structure is composed of five domains, organized in a triangular pyramid shape (Figure 3). At the apex of the pyramid are the N-terminal zinc binding (ZBD) and the stalk (S) domains (Figure 3B). Domains 1A and 2A present the RecA-like structure typical of helicases, with the catalytic site located at their interface. The catalytic pocket of the enzyme is located between the two RecA domains 1A and 2A and include the six residues K288, S289, D374, E375, Q404, and R567 (Figure 3B). Interestingly, the unwinding activity of SARS-CoVNsp13 is stimulated by its interactions with the RNA dependent RNA polymerase (RdRP, Nsp12), the main enzyme responsible for CoV RNA polymerisation, with this interaction mediated by ZBD and 1A domains (Figure 3B) (Jia et al., 2019). The high sequence conservation of Nsp12 and Nsp13 in all CoVs (Supplementary Table 1) suggests their association as a common feature across CoVs (Jia et al., 2019).

### Nsp14, a Key Enzyme Involved in mRNA Proofreading and Final Capping

Nsp14of SARS-CoV-2 is a peculiar bi-functional enzyme. It is composed of two different functional domains: an N-terminal exoribonuclease (ExoN) domain and a C-terminal N7-guanine MTase domain (N7-MTase). The ExoN domain of Nsp14 hydrolyses single-stranded and double-stranded RNAs and is critical for proofreading function in coronaviruses (Denison et al., 2011; Ogando et al., 2019), a property that is missing in other RNA viruses. Due to the presence in the ExoN domain of three conserved motifs, motif I (DE), II (E), and III (D), Nsp14 is classified as a “DEED outlier” among DEDD exonucleases (Ma et al., 2015; Ogando et al., 2019). Knockout mutants of ExoN in Murine Hepatitis Virus (MHV), SARS-CoV, Alphacoronaviruses HCoV-229E, and Transmissible Gastroenteritis Virus (TGEV) were shown to display defects in the synthesis of genome and sub-genome length RNAs and a reduction in replication efficiency, thus emphasizing the supporting role of Nsp14 in CoV replication (Minskaia et al., 2006; Eckerle et al., 2007, 2010). In this context, Nsp14, through its ExoN domain, is part of the RNA replication machinery.

The carboxy-terminal region of Nsp14, containing N7-guanine MTase activity, instead plays a key role in capping of viral both genomic and sub-genomic mRNAs. The cap synthesis starts with the hydrolysis of the 5′ end of a nascent RNA by the RNA 5′-triphosphatase Nsp13 to yield pp-RNA (Ivanov and Ziebuhr, 2004). Subsequently, a still unknow GTase transfers a GMP molecule onto the pp-RNA to yield Gppp-RNA. The cap structure is then methylated at the N7 position by the N7-MTase domain of Nsp14 (Chen J. Y. et al., 2009; Chen Y. et al., 2013). In addition to being important for the stability of mRNAs, the cap structure is essential to avoid the host immune response and allows the ribosomal complex to recognize mRNAs to ensure their efficient translation (Decroly et al., 2012).

**Figure 4:**
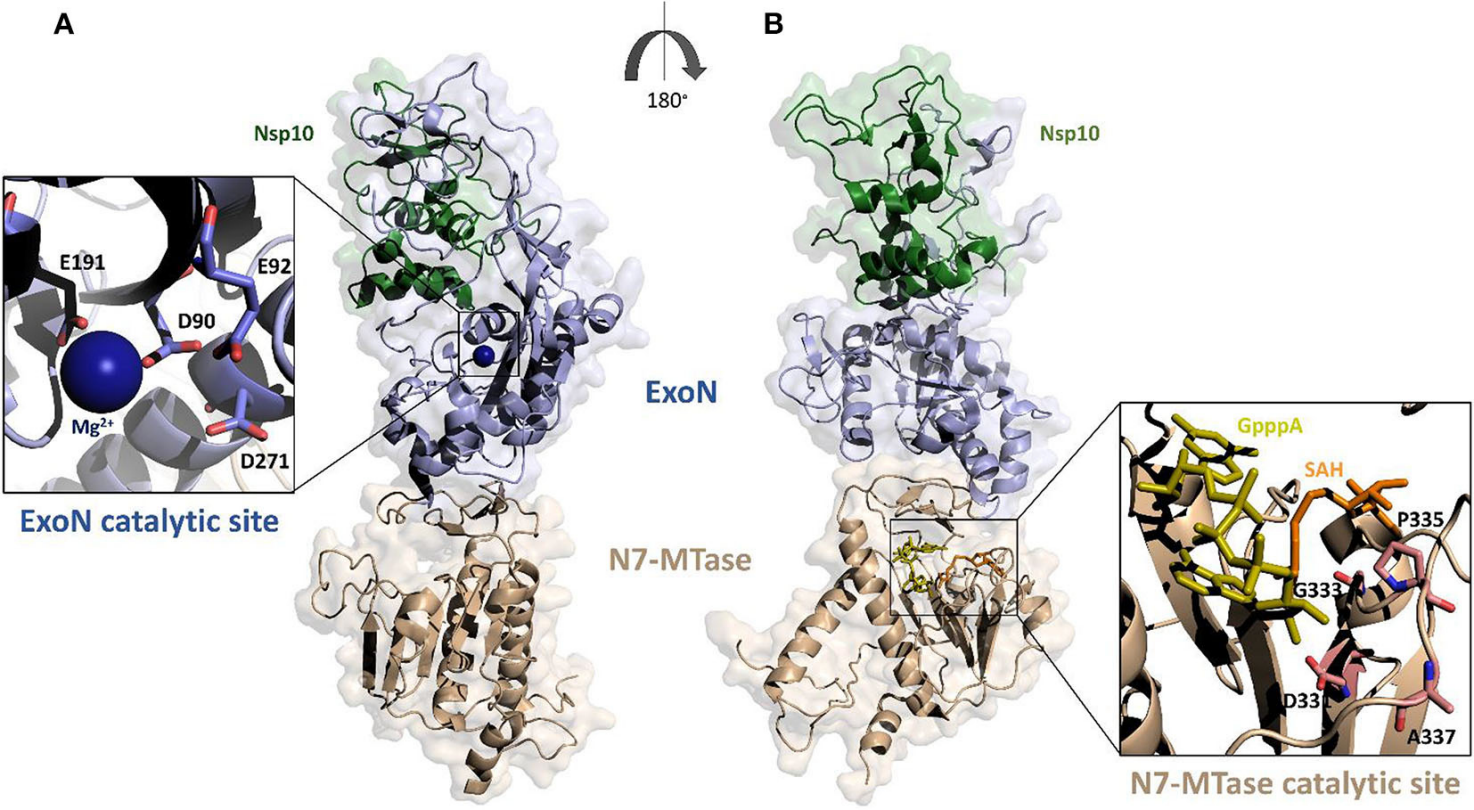
Surface representation of the homology model of SARS-CoV-2 Nsp14-Nsp10 complex. The model was calculated using MODELER and the structure of SARS-CoV Nsp14-Nsp10 complex as a template (PDB code 5c8s, region 1-131 of Nsp10 and 1-525 of Nsp14); **(A,B)** panels show 180° views, to highlight catalytic sites of the two functional domains of Nsp14. The ExoN domain is shown in light blue and N7-MTase domain in wheat color. Zooms of the catalytic sites are shown (cartoon and stick representations) in the insets. The functional ligands of the N7-MTase domain (SAH, the demethylated form of SAM and GpppA), are represented as sticks, respectively in orange and olive (Ma et al., 2015).

Based on the crystal structure of SARS-CoV Nsp14-Nsp10 complex (PDB code: 5C8S), we have generated, using Modeler, a homology model of the complex between Nsp14 and Nsp10 of SARS-CoV-2 (Figure 4) (Ma et al., 2015; Ferron et al., 2018). In this heterodimer, the co-factor Nsp10 forms numerous interactions with the ExoN domain of Nsp14 (Bouvet et al., 2014). In particular, the first 25 residues of Nsp14 form a clamp that accommodates the first 10 residues of Nsp10. This structural relationship is the key to the ExoN activity. Indeed, this tight interaction between Nsp10 and the N-terminus of Nsp14 allows the exonuclease active site to adopt a stable and highly active conformation. These structural findings, together with experimental evidence, underline that the interaction with Nsp10 strongly influences the nucleolytic activity of Nsp14 (Bouvet et al., 2012). Consistently, as shown by SAXS experiments, in the absence of Nsp10, Nsp14 from SARS-CoV shows large conformational changes at its N terminus, which affect the overall structural architecture of the ExoN domain, leading to weak ExoN activity (Ferron et al., 2018).

The ExoN domain of Nsp14 is essentially composed of a central twisted β-sheet which is formed by five β-strands flanked by α-helices (Ma et al., 2015; Ferron et al., 2018). This architecture is typical of exonucleases of the DEDD superfamily, like RNase T from *E.coli* and RNase AS from *M. tuberculosis* (Derbyshire et al., 1991; Romano et al., 2014, 2015). The catalytic tetrad was identified using the structural alignment with SARS-CoV Nsp14 and include the DEED residues Asp90, Glu92, Glu191, and Asp272 (Figure 4A).

A flexible hinge region connects the ExoN domain with the N7-MTase domain of Nsp14. This region, consisting of a loop and three strands, is highly conserved across CoVs and permits significant movements of the two domains (Figure 4) (Ma et al., 2015; Ogando et al., 2019). As mentioned before, the N7-MTase domain participates in mRNA capping by adding a methyl at the N7 position of the guanosine of the mRNA cap structure. To complete this enzymatic reaction, it uses the co-enzyme S-Adenosyl Methionine (SAM) as methyl donor (Figure 4). The N7-MTase domain does not belong to any of the classes of SAM-dependent MTases (Schubert et al., 2003; Byszewska et al., 2014; Chouhan et al., 2019) and shows a non-canonical MTase fold different from the Rossmann fold of the virus RNA MTase (Rao and Rossmann, 1973; Ferron et al., 2018). Indeed, whereas the canonical fold is formed by a seven-strand β-sheet with at least three parallel α-helices on each side, Nsp14 contains a central β-sheet composed by only a five-strand (Figure 4B) (Rao and Rossmann, 1973). The N7-MTase domain presents two clusters of residues key for its enzymatic activity; the first cluster is a canonical SAM-binding motif I (DxGxPxG/A), including Asp331, Gly333, Pro335, and Ala337 (Figure 4B). A second cluster forms a pocket that holds the GTP of the mRNA cap structure close to the methyl donor SAM.

## A Structural Overview of Human DDX Helicases Involved in COV Interaction

**Figure 5:**
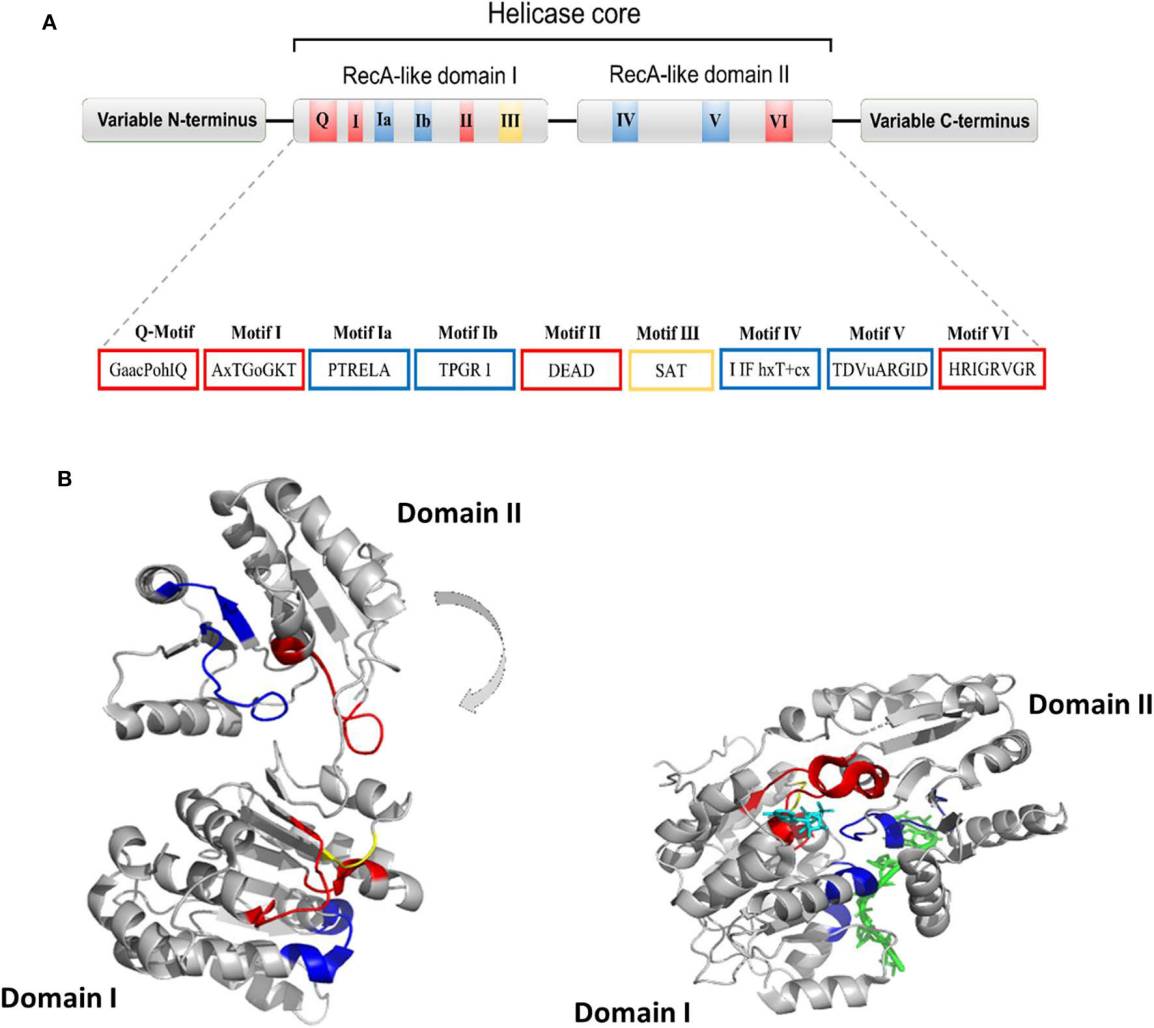
**(A)** Typical domain organization of DDX helicases. The motifs are shown with colors according to their primary function (red, ATP binding and hydrolysis; yellow, coordination between NTP- and RNA-binding sites; blue, RNA binding). **(B)** Cartoon representation showing domain movements in DDX helicases upon RNA binding of the DEAD-box protein eIF4AIII without RNA (left) and in complex with RNA (right). An RNA fragment and AMPPNP are shown in stick representation in green and in cyan, respectively.

Human DDX helicases belong to the DEAD-box protein family, which is the largest family of the superfamily 2 (SF2) helicases (Byrd and Raney, 2012). They share a highly conserved helicase core consisting of two RecA-like domains (D1 and D2) connected via a flexible linker region (Hilbert et al., 2009). These two domains, also known as DEAD domain and helicase domain, are characterized by the presence of nine conserved motifs, Q, 1, 1a, 1b, II, III, IV, V, and VI, and the common tetrapeptide (Asp-Glu-Ala-Asp) in motif II (Figure 5A). In many DEAD box helicases, the D1D2 domains are linked to distinct flanking regions or ancillary domains, ranging from a few to several hundred amino acids in length (Rudolph and Klostermeier, 2015). These variable N-terminal and C-terminal domains contribute to the functional diversity of this protein family, and direct individual helicases to their targets via protein or RNA interaction or by modulating the activity of the helicase core (Del Campo and Lambowitz, 2009; Mallam et al., 2011; Ferrage et al., 2012; Rudolph and Klostermeier, 2015). Structural studies on DDX helicases, reported in Tables 2, 3, are mainly focused on the helicase core or isolated ancillary domains, with few structures of full-length helicases. Nevertheless, the conserved patch in the helicases core provides important insights on their activity. In DDX proteins, the two RecA-like domains are both responsible for RNA binding and ATP hydrolysis (Hilbert et al., 2009). Specifically, the conserved motifs Q, I (P-loop), II, and VI participate in ATP binding and hydrolysis; motif III is responsible for coupling NTP hydrolysis to nucleic acid unwinding, and motifs Ia, Ib, IV, and V are involved in RNA binding (Figure 5) (Hilbert et al., 2009).

**Table 2:** Structural similarity of SARS-CoV-2 Nsp13 with human DDX helicases, as computed by DALI.

	**PDB code**	**Z score**	**Seqid (%)**	**Rmsd (Å)**	**Length of alignment**
DDX20	2oxc	9.8	12	3.1	149
DDX53	3iuy	9.5	10	3.0	213
DDX10	2pl3	9.3	14	3.1	147
DDX47	3ber	9.1	10	3.0	146
DDX52	3dkp	9.0	11	3.3	148
DDX18	3ly5	8.9	8	3.4	150
DDX17	6uv1	8.7	11	3.6	200
DDX5	4a4d	8.4	11	3.4	155
DDX3X	6o5f	7.8	8	3.8	155
DDX41	2p6n	7.7	11	3.0	131
DDX25	2rb4	7.1	8	3.2	128

**Table 3:** Structures of DDX helicases.

**Protein**	**PDB code**	**Sequence information/_**residue**_**	**Ligands**
DDX1	4XW3	SPRY domain	None
DDX3X	2JGN	Domain II _409−580_	None
	4PX9	NTE and Domain I _135−407_	ADP
	4PXA	NTE and D1D2 core _135−582_ (D354V)	ADP, PO_4_
	2I4I	D1D2 core _18−582_	AMP
	5E7I, 5E7M, 5E7J	NTE and D1D2 core _133−584_	None – AMPPNP – AMP
	6O5F	NTE, CTE and D1D2 core _132−607_	23-bp dsRNA
	6CZ5	NTE, CTE and D1D2 core _132−607_ (S228)	AMP-acrylamide
	3JRV	N-terminal peptide _71−90_	Protein K7 (*Vaccinia virus*)
	4O2C, 4O2E, 4O2F	N-terminal peptides SL9 NAc, SL9, HL8	HLA-B*3901 (HLA class I histocompatibility antigen, B-39 alpha chain)
DDX5/p68	4A4D	Domain I _52−304_	None
	3FE2	Domain I _68−307_	ADP, SO_4_
DDX6/p54	1VEC	Domain I _94−299_	Zn^+2^, C_4_H_6_O_6_
	2WAY, 2WAX	Domain II _296−483_	EDC3 FDF peptide
	4CRW	Domain II _307−483_	CNOT1 MIF4G domain (fragment)
	6F9S	Domain II _301−469_	LSM14 peptide, SO_4_
	6S8S	Domain II _295−483_	EDC3 FDF peptide, PO_4_
	4CT5, 4CT4, 5ANR	D1D1 core _95−469_	None - CNOT1 MIF4G domain, Mg^+2^ - 4E-T/CNOT1
DDX10	2PL3	Domain I _47−280_	ADP, Mg^+2^
DDX17	6UV0	D1D2 core _111−556_	Mg^+2^
	6UV1	D1D2 core _111−556_	rU10 RNA, ADP, Mg^+2^
	6UV2	D1D2 core _111−556_	pri-125a-oligo1RNA, ADP, Mg^+2^
	6UV3	D1D2 core _111−556_	pri-125a-oligo2 RNA, ADP, Mg^+2^
	6UV4	D1D2 core _111−556_	pri-18a-oligo1 RNA, ADP, Mg^+2^
DDX18	3LY5	Domain I _149−387_	PO_4_, Mg^+2^
DDX19B	3FHC	Domain I _68−302_	Nup214
	3FHT	D1D2 core _68−479_ (ΔN67)	AMPPNP, U10 ssRNA, Mg^+2^
	3FMO - 3FMP	NTE and Domain I _1−300_ - Full length	Nup214, ADP
	3EWS, 3G0H	D1D2 core _54−475_ (ΔN53)	ADP - AMPPNP, U_10_ RNA, Mg^+2^
	6B4K - 6B4J - 6B4I	D1D2 core _54−479_ (ΔN53)	AMPPNP, Mg^+2^ - AMPPNP/Gle1^CTD^-Nup42^GBM^, Mg^+2^ - ADP/Gle1^CTD^-Nup42^GBM^
DDX20	2OXC	Domain I_41−268_	ADP
	3B7G	Domain I_41−268_	AMPPNP
DDX21	2M3D	GUCT domain	None
DDX23/Prp28	4NHO	D1D2 core _338−820_ (ΔN337)	SO_4_, Hg^+2^
	3JCR	Full length	U4/U6.U5 tri-snRNPspliceosomalcomplex
	6AH0	Full length	Precursor of pre-catalytic spliceosome
	6QX9	Full length	Fully assembled pre-catalytic spliceosome
	6QW6	Full length	U4/U6.U5 spiceosomal tri-snRNPcomplex
DDX25	2RB4	Domain II _307−479_	SO_4_, Zn^+2^
DDX41	2P6N	Domain II _402−569_	None
	5GVR - 5GVS	Domain I _169−402_ closed form - open form	None
	5H1Y	Domain I _153−410_	SO_4_, Mg^+2^
DDX47	3BER	Domain I _5−230_	AMP, PO_4_
DDX48/eIF4AIII	2HXY	D1D2 core _23−411_	None
	2HYI - 2XB2 - 3EX7	D1D2 core _1−411_	ECJ (Exon junction complex), RNA, AMPPNP, Mg^+2^ – UPF3b ECJ, AMPPNP, Mg^+2^ – ECJ transition state, ADP, Mg^+2^
	4C9B	D1D2 core _1−411_	CWC22, PO_4_
	2J0Q, 2J0S	D1D2 core _2−411_	ECJ, AMPPNP, Mg^+2^
	2J0U	D1D2 core _39−411_	CASC3
	5YZG, 5MQF, 5XJC, 6ICZ, 6QDV	D1D2 core _1−411_	C complex, step I - C complex, step II – P complex (human spliceosome)
DDX50	2E29	GUCT domain	None
DDX52	3DKP	Domain I_139−381_	ADP, Mg^+2^
DDX53	3IUY	Domain I _204−430_	AMP
DDX58/RIG-1	2LWD, 2LWE	CARD 2 domain _95−190_ WT and T170E variant	None
	4NQK - 4P4H	CARD 1 and 2 domains _1−200_	Ubiquitin - CARD^MAVS^, UBA-52
	2RMJ - 3NCU	CTD _792−925_	None - 12bp blunt-end 5′-pp dsRNA, Zn^+2^
	2QFB - 2QFD - 3LRN - 3LRR - 3OG8	CTD _802−925_	Zn^+2^ - Hg^+2^ - 14bp GC rich 5' ppp dsRNA, Zn^+2^ - 12bp AU rich 5′ ppp dsRNA, Zn^+2^ - 14bp blunt-end dsRNA, Zn^+2^
	4ON9	D1D2 core _230−793_	SO_4_
	5E3H, 6GPG	CTD and D1D2 core _232−925_ (ΔCARDs) - C268F	14bp dsRNA, ADP, Mg^+2^, Zn^+2^ - 14bp dsRNA, Mg^+2^, Zn^+2^
	2YKG, 4BPB, 3ZD6, 3ZD7	CTD and D1D2 core _230−925_ (ΔCARDs)	5′OH-GC10 dsRNA, Zn^+2^, SO_4_ - 5′OH-GC10 dsRNA, Zn^+2^ - 5′OH-GC10 dsRNA, ADP, Mg^+2^, Zn^+2^
	4AY2, 5F9F, 5F9H, 5F98	CTD and D1D2 core _232−925_ (ΔCARDs)	5′ppp 8-bp HP RNA, ADP, Mg^+2^, Zn+2 - 5'OH HP RNA, Mg^+2^, Zn^+2^ - 5'ppp HP RNA, Mg^+2^, Zn^+2^ - Cap-0 HP RNA

*NTE (N-terminal extension), CTE (C-terminal extension),CTD(C-Terminal regulatory Domain), CARD (Caspase recruitment domain)*.

Comparative studies on the helicase core highlighted a structural mechanism of open-close conformation of the two RelA-like domains, suggesting a link between the binding of ATP and activation of the RNA binding site (Theissen et al., 2008; Schutz et al., 2010). In the absence of a ligand, the D1 and D2 domains are separated and more flexible, resulting in an open conformation (Figure 5B). Upon ATP and RNA binding, they approach each other in a more compact arrangement, thus forming a cleft which aligns the catalytic site for ATP hydrolysis (Figure 5B). In particular, the P-loop and motif II coordinate the nucleotide phosphates and the magnesium ion, whereas residues of the Q-motif bind and recognize the adenine moiety (Schutz et al., 2010) (Figure 5B). The domain closure mechanism is consistent with biochemical studies, indicating that ATP and nucleic acid binding is highly cooperative in DDX proteins, most of which are unable to bind or hydrolyze ATP without RNA (Jankowsky and Fairman, 2007; Theissen et al., 2008). Importantly, in all DEAD-box protein structures, conserved amino acids make contact exclusively with the sugar-phosphate backbone of the RNA, including several interactions with the 2′-OH groups (Jarmoskaite and Russell, 2011). This finding explains why RNA binding is not sequence specific but DDX can still distinguish RNA from DNA.

### The Two Homologs DDX3X and DDX5

**Figure 6:**
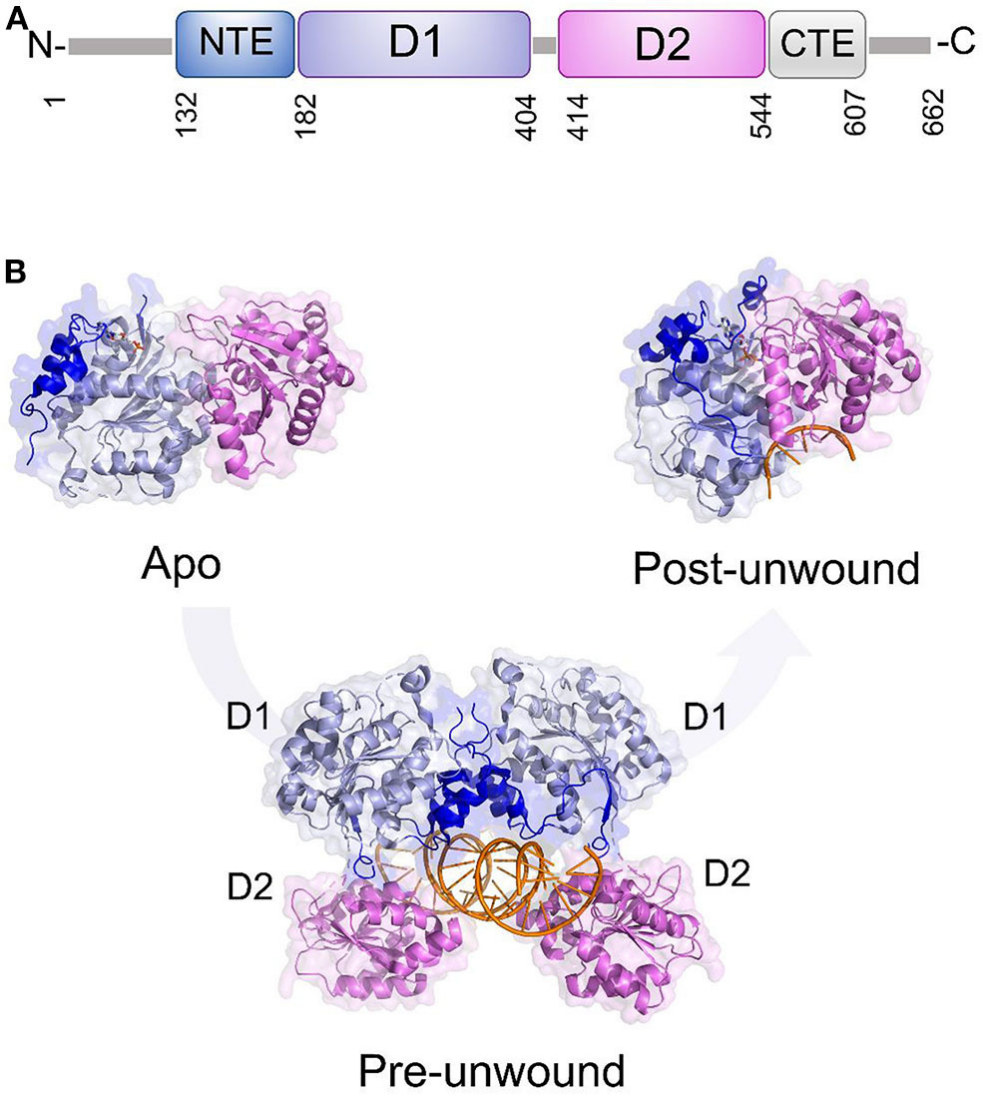
DDX3X as a model for dsRNA unwinding mechanism by DDX helicases. **(A)** Domain organization and **(B)** cartoon representation of DDX3X in its proposed functional states, including the apo and pre-unwound states (pdb codes 5e7i and 6o5f, respectively), and its post-unwound state (pdb code: 2db3, ortholog from *Drosophila melanogaster*).

Like all DDXs, DDX3X contains a helicase core composed of two Rec-Alike domains, constituting the D1D2 core. This core is flanked by N- or C-terminal unstructured tails (Figure 6A). Despite the lack of structural information on the N (NTE) and C terminal tails (CTE), evidence has accumulated showing that NTE contains a nuclear export sequence and is key for DDX3X nuclear export (Heaton et al., 2019). On the other hand, CTE is essential for DDX3X oligomerisation, a process which is required for optimal helicase activity (Putnam et al., 2015).

As for the D1D2 core, DDX3X is one of the most structurally characterized DDX helicases. The crystal structure D1D2 core of DDX3X has been reported in several functional states: the apo state,; the pre-unwound state, consisting of a complex with a 2-turn dsRNA; and a post-unwound state, in which DDX3X is complexed with a single strand RNA (Figure 6) (Song and Ji, 2019). These studies have shown that the binding of dsRNA to apoDDX3X triggers dramatic conformational changes of the enzyme to form the pre-unwound DDX:dsRNA:DDX complex (Figure 6B). This structure, also confirmed in solution, resembles a clamp that locks dsRNA among four RecA-like domains (Figure 6B), with one D1D2 core mainly binding RNA Strand 1 and the other D1D2 core binding RNA Strand 2 (Song and Ji, 2019). In the transition from a pre-unwound to a post-unwound state, RNA unwinding is driven by ATP hydrolysis and finally leads to a monomeric complex of DDX3X and single strand RNA (Figure 6B). In this unwinding model, the two dsRNA-bound D1D2 cores undergo dramatic conformational changes upon the binding of MgATP and thus unwind dsRNA (Figure 6). This mechanism has been proposed as a general unwinding mechanism of DDX helicases (Song and Ji, 2019). DDX3X has a confirmed role in anti-viral immune signaling pathways leading to type I IFN induction (Schroder et al., 2008; Soulat et al., 2008; Gu et al., 2013, 2017). Notably, DDX3X has already been validated as a target for broad-spectrum antiviral molecules against a number of RNA viruses (HIV, HCV, DENV, and WNV).

**Figure 7:**
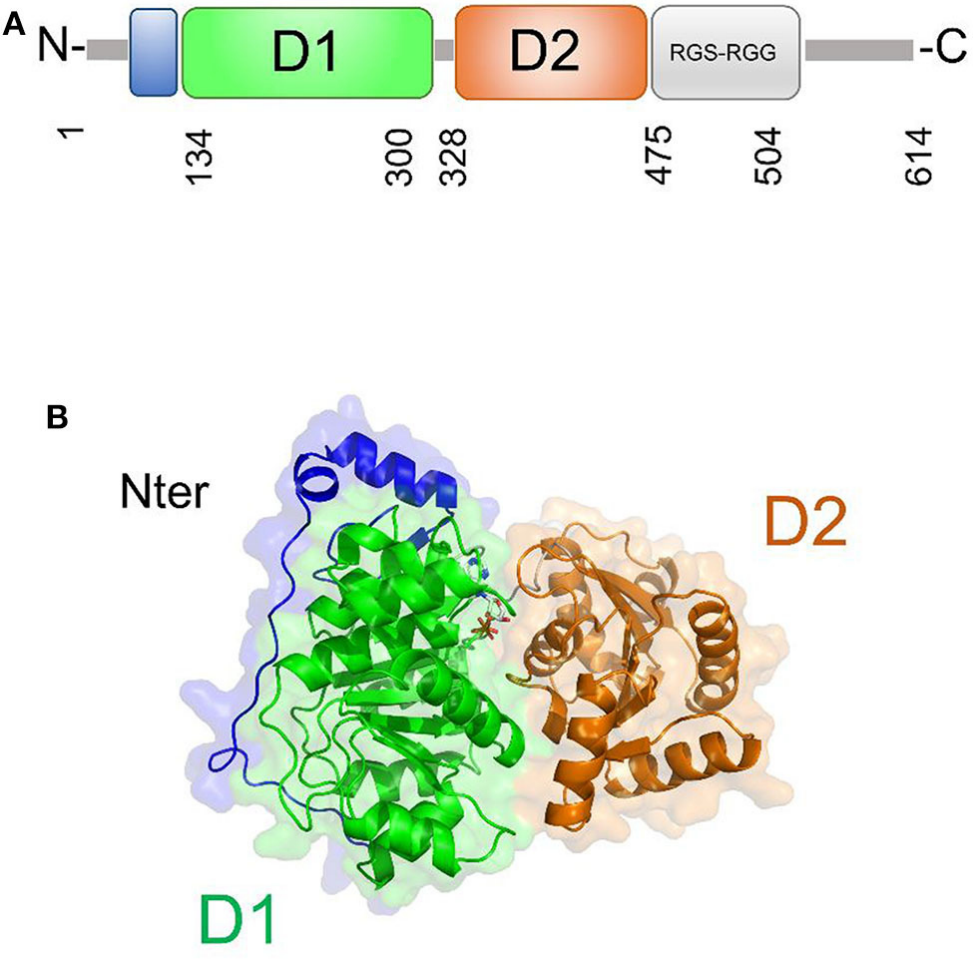
DDX5 structural organization. **(A)** Domain organization and **(B)** cartoon representation of the homology model of DDX5, obtained using Modeler and the structure of DDX17 (pdb code 6uv1, covered region 35–476, seqid 83.7%) as a template.

DDX5 plays fundamental roles in transcriptional regulation and in viral replication (Cheng et al., 2018). Like other DEAD-box helicases, DDX5 shares the modular domain architecture with the nine conserved motifs constituting the core region (Figure 7). Moreover, DDX5 helicase contains a Arg-Gly-Ser-Arg-Gly-Gly (RGS-RGG) motif and an Ile-Gln (IQ) motif, which is localized in the C-terminal region and can act either as RNA-binding site or protein interaction module (Rajyaguru and Parker, 2012) (Figure 7). Indeed, there is evidence that DDX5 may interact with other DEAD-box helicases, such as DDX3X and its close homolog DDX17; an interaction between DDX5 and DDX3X was proposed as a novel combined mechanism of action for DEAD-box RNA helicases involved in RNP remodeling and splicing (Ogilvie et al., 2003; Choi and Lee, 2012).

The only structural information available for DDX5 include domain I and part of the variable N-terminal region (Schutz et al., 2010) (Table 3). However, DDX5 shares a high sequence identity with DDX17 (83.7%, region 35-476), whose structure is known (Ngo et al., 2019). Therefore, we computed the homology modeling structure of a major portion of DDX5, including an N-terminal region of unknown function and the D1D2 core (Figure 7A). In this structure, the N-terminal region crosses the entire length of the D1 domain, reaching the RNA substrate binding region (Figure 7). In DDX17, the N-terminal extension can modulate its ATPase and unwinding activities (Ngo et al., 2019). The high sequence similarity of DDX5 with that of DDX17 suggests a similar autoregulatory role of the N-terminal region in controlling the ATPase activity, through the observed intramolecular interaction between N-terminal tail and the D1 domain (Ngo et al., 2019) (Figure 7). As for the D1D2 core, structural similarity analysis by DALI server shows that DDX5 D1D2 (PDB entry 3fe2) possesses high structural similarity with DDX3X, as well as with DDX17 and DDX41 (z-scores 31.4, 35.9, 33.5, respectively), thus indicating similar roles for all these DDX.

### DDX1, a Structurally Unique DDX

**Figure 8:**
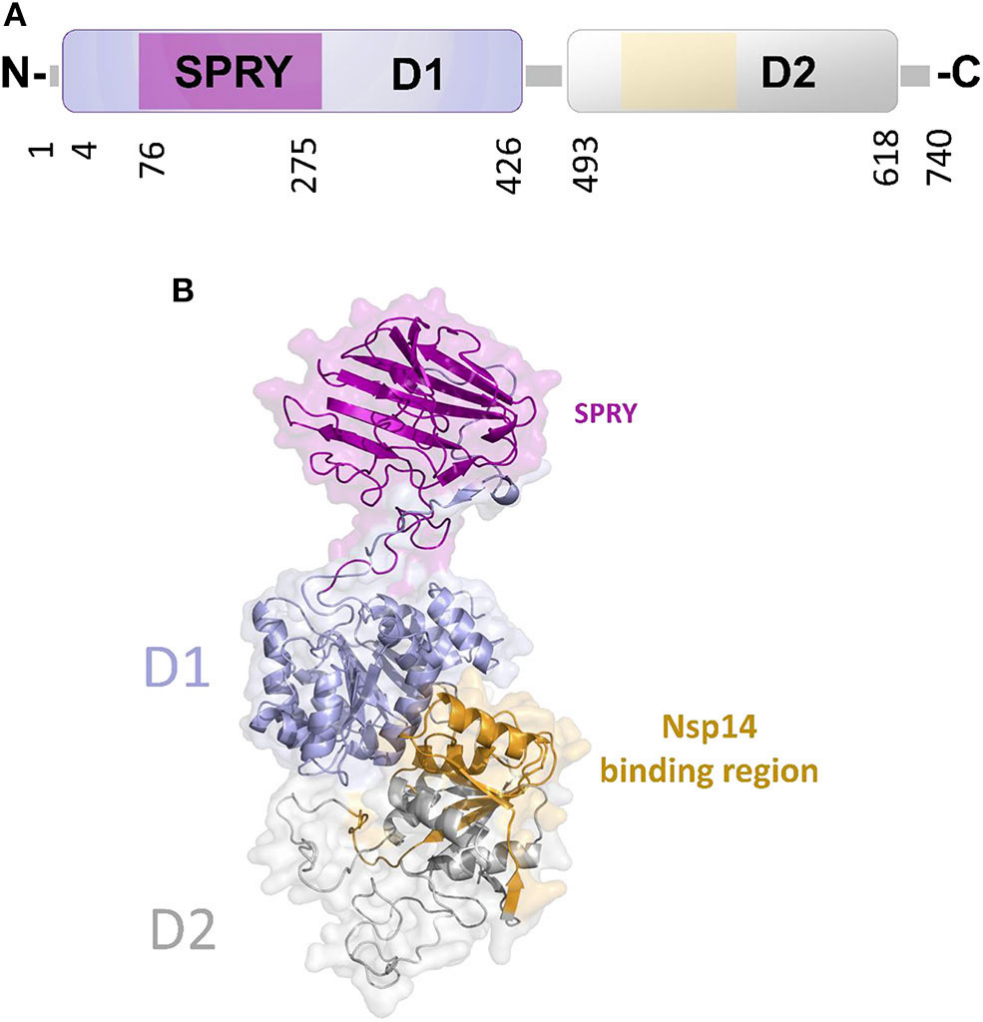
The unique structure of DDX1. **(A)** Domain organization according to PFAM. **(B)** Cartoon representation of the homology model of DDX1, computed using Modeler and the structures of DDX Helicase CshA (PDB code 5ivl, coverage 7–75; 276–674, 35% seqid) and of DDX1 SPRY domain (PDB code 4xw5, coverage 76–275, seqid 100%) as templates. The Nsp14 binding region in the helicase domain of DDX1 is highlighted in yellow.

In DEAD-box helicases, different insertions, N- or C-terminal appendages, or additional domains to the standard modular architecture of RecA-like domains may be responsible for the diversity of DEAD-box protein functions. As an example, DDX1 is unique among DEAD-box proteins because of a domain insertion in the amino-terminal helicase domain (Kellner et al., 2015). This extra domain, denoted as SPRY (SPla and the RY anodine Receptor), is inserted between the P-loop and the conserved motif Ia of the RecA domain D1 (Figure 8A). The SPRY domain is the sole structural information hitherto available for DDX1 and shows a typical β-sandwich fold, with a variability in the loop regions (Kellner and Meinhart, 2015). However, using structural information from its homologous structures, we computed a homology model of the entire DDX1, for a better description of its structural and binding properties (Figure 8B). In this structure, the SPRY domain protrudes from the structure of the helicase and is located on the opposite side of the enzyme catalytic site. Consistently, SPRY is a protein interaction module implicated in important biological pathways, including innate immunity (D'Cruz et al., 2013), regulating DDX1 assembly into multiprotein complexes. However, the interacting partners of SPRY remain unknown, as do the molecular determinants of binding specificity.

## Involvement of COV Proteins in DDX Hijacking

### The N Protein Is a Central Hub for DDX Interactions

The multifunctional role of the N protein in the virus life cycle, from regulation of replication and transcription and genome packaging to modulation of host cell processes, strongly relies on interactions with host cell proteins (Emmott et al., 2013). Consistently, this protein has been shown to be involved in interactions with multiple host proteins in IBV-CoV and SARS-CoV, including proteins of the DDX family (Emmott et al., 2013) (Table 1). An interaction of N protein has also been observed directly in SARS-CoV-2 with the helicase DDX21 (Gordon et al., 2020). However, the effect of this interaction on viral replication is currently unknown.

Among DDX proteins interacting with the N protein, DDX3X is massively involved in immune reactions against the viral infection. In particular, it is involved in the interferon (IFN)-mediated innate immune cascade (Gu et al., 2013) and is a positive regulator of the TBK/IKKε signaling cascade, as it interacts with and acts as a substrate of the TBK1 and IKKε kinases, activating the IRF3 transcription factor (Brai et al., 2020a). DDX3X is also recruited to the IFN promoter through interaction with IRF3 itself (Gu et al., 2013). In addition, DDX3X interacts with IPS-1 and TRAF3, components of the RIG-I sensor complex, for the recognition of viral RNA and activation of the IFN response (Oshiumi et al., 2010; Gu et al., 2017) and modulates the NF-kB inflammatory response through activation of the IKKβ/PPA2C complex (Wang et al., 2017). Therefore, it is possible to hypothesize that the N protein plays a role in modulating the immune response mediated by DDX3X and that binding of N to DDX3X results in the inhibition of these antiviral pathways. Indeed, a similar mechanism acting on DDX3X has been already demonstrated for other viruses (Schroder et al., 2008).

DDX1 and DDX21 were also shown to belong to the interactome of the N protein in IBV-CoV (Emmott et al., 2013) (Table 1). Interestingly, these two DDX proteins have been shown to interact with each other to form a large complex with another RNA helicase, DHX36, and with the adaptor molecule TRIF, forming a sensor of viral dsRNA in dendritic cells (Zhang et al., 2011). This DDX1-DDX21-DHX36 complex uses DDX1 to bind dsRNA and DDX21, and DXH36 to bind TRIF, to activate the NF-kB pathway and type I IFN responses in dendritic cells (Zhang et al., 2011). In this context, the DDX1-DDX21-DHX36 complex helps the innate immune system to detect viral infection by sensing viral nucleic acids cells (Zhang et al., 2011). Targeting of this complex by the N protein plays a role in immune evasion, possibly interfering with the formation of the sensor complex or by inhibiting the recognition of dsRNA by DDX1. However, research studies are still needed to clarify this aspect, as details of these interactions are currently unknown.

**Figure 9:**
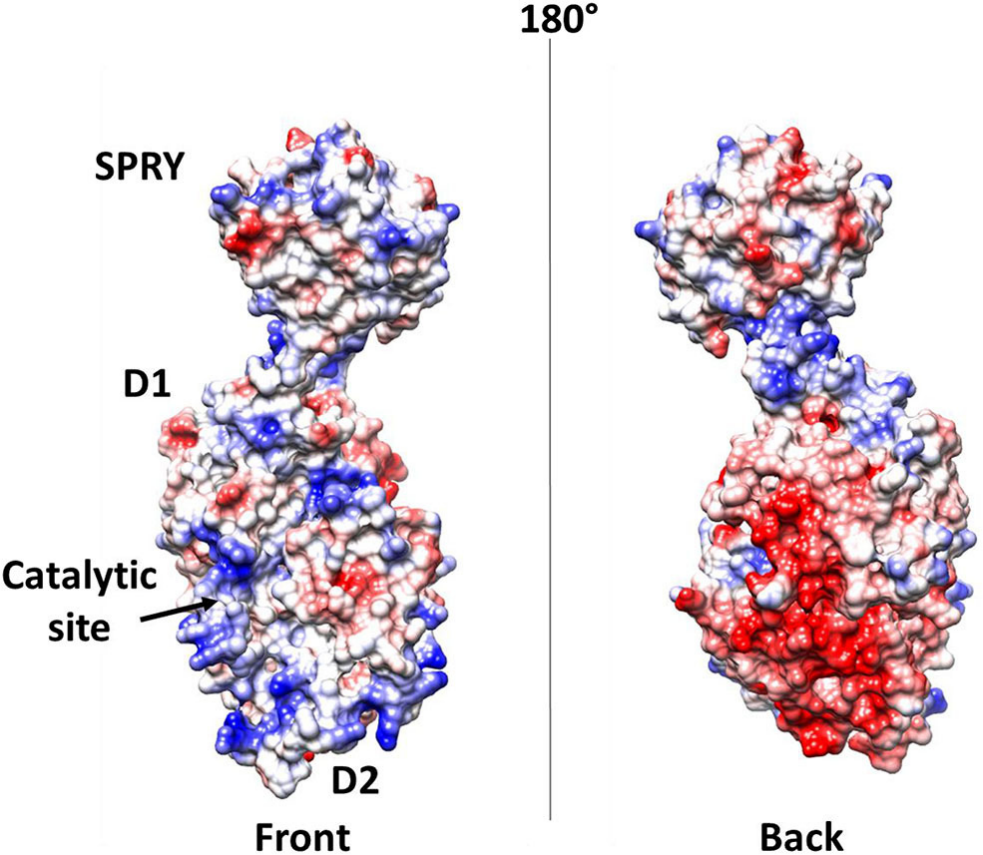
Electrostatic potential surface of DDX1. Left and right panels show two opposite sides of the molecule.

Further elements are known on the interaction of DDX1 with the N protein (de Haan and Rottier, 2005; McBride et al., 2014; Wu et al., 2014). As discussed above, the N protein is a highly basic protein with phosphorylation modifications, predominantly found in serine residues clustered within the central Ser-arginine (SR)-rich motif located in the LKR linker, which connects the NTD and CTD domains (Wu et al., 2009). This crucial protein also regulates the discontinuous transcription of sub-genomic mRNAs, since its depletion reduces the synthesis of subgenomic mRNA, but not of genomic RNA (Zuniga et al., 2010). Interestingly, the phosphorylation of the N protein by host kinase GSK-3 allows the recruitment of DDX1 helicase, as DDX1 specifically interacts with the N protein of JHMV phosphorylated at Ser197 (pS197-N) belonging to the LKR linker (Wu et al., 2014). In this process, DDX1 plays a role in the regulation of viral RNA transcription by increasing the synthesis of longer viral RNAs. Indeed, DDX1 knockdown markedly reduces the synthesis of longer RNAs of JHMV but minimally reduces the synthesis of shorter sub-genomic mRNA (Wu et al., 2014). Consistently, overexpression of the wild-type, but not of an inactive mutant of DDX1, increases the synthesis of longer viral RNAs, showing that the effect of DDX1 on viral RNA synthesis is linked to its enzymatic function (Wu et al., 2014). This evidence suggests that the involvement of host DDX1, through interactions with the phosphorylated N protein, is a unique viral strategy to support the transition from discontinuous to continuous transcription (Wu et al., 2014). Mechanistic details of this regulatory interaction are unknown. However, the key involvement of phosphorylated serine residues in DDX1 binding, as discussed above, suggests an electrostatic interaction between the LKR linker and the highly charged DDX1 surface to regulate its function (Figure 9).

### Nsp14 and DDX1, a Molecular Liaison to Repress Viral Induced IFN-β Immune Reaction

**Figure 10:**
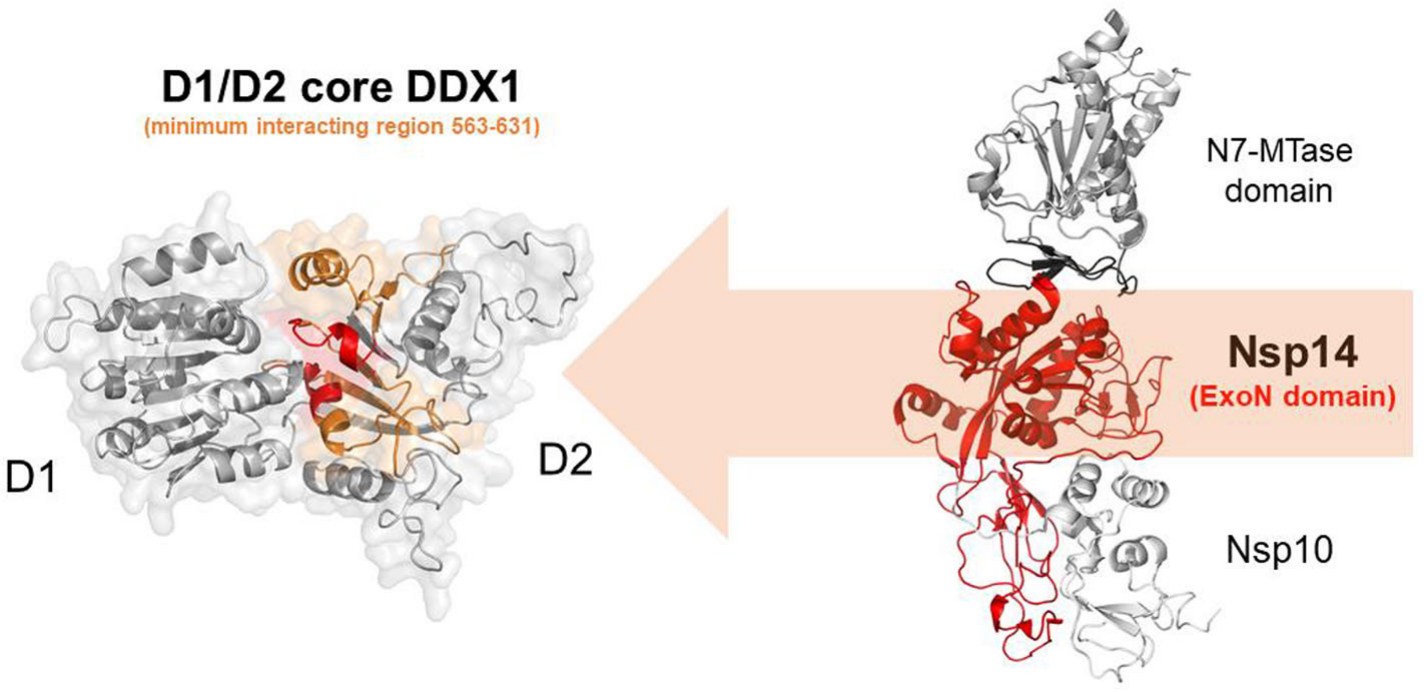
Nsp14-mediated DDX1 hijacking by SARS-CoV-2. Cartoon representation of DDX1 D1D2 core (left)and Nsp14/Nsp10 (right) obtained by homology modeling. Nsp14 and DDX1 interaction involves the C-terminal region of DDX1 (orange) with motifs IV and V (red), and the ExoN domain of Nsp14 (red).

In addition to the N protein, Nsp14 has also been shown to interact with DDX1 in both IBV-CoV and SARS-CoV (Xu et al., 2010; Zhou et al., 2017). Importantly, this interaction enhances coronavirus replication, as confirmed by manipulating DDX1 expression, either by small interfering RNA-induced knockdown or by overexpression of a mutant DDX1 protein (Xu et al., 2010). As previously explained, Nsp14 is involved in replication and transcription of the viral genomic and subgenomic RNAs. Therefore, a possibility for the observed enhancement of CoV replication is that DDX1 binding to the Exo domain may facilitate this process. Structurally, the interaction between Nsp14 and DDX1 involves the C-terminal portion of the DDX1 helicase, containing motifs V and VI, and the N-terminal Exo domain of Nsp14, although details of the interaction are currently unknown (Xu et al., 2010) (Figure 10). Further studies are also needed to elucidate the effect of DDX1 interaction with Nsp14 on the exoribonuclease and N7-guanine MTase activities catalyzed by Nsp14.

It is interesting to note that, in IBV-infected cells, DDX1 relocates from the nucleus to the cytoplasm with a predominant staining pattern in the viral RNA replication site, suggesting that the interaction of Nsp14 could alter the subcellular localization of DDX1 (Xu et al., 2010). Also, because phosphorylation protein N does not affect the interaction between DDX1 and Nsp14, it is likely that the two interaction patterns follow independent roots (Wu et al., 2014).

Interestingly, an opposite effect of the interaction between Nsp14 and DDX1 was observed in TGEV, with DDX1 showing antiviral activity against TGEV replication (Zhou et al., 2017). Nsp14 was shown to induce a DDX1-dependent IFN-β production via NF-κB pathway in TGEV infection (Zhou et al., 2017). Indeed, knockdown of DDX1 significantly decreased Nsp14-induced IFN- β production and NF- κ B activation (Zhou et al., 2017). As discussed above, DDX1 and helicases DDX21 and DHX36 are involved in sensing viral dsRNA and inducing IFN-β production. Therefore, Nsp14 of TGEV may be recognized as a Pathogen Associated Molecular Patter molecule (PAMP) by the DDX1 portion of the DDX1-DDX21-DHX36 viral sensor (Zhang et al., 2011). This antiviral effect of DDX1 in TGEV witnesses a different regulation of CoVs compared to other bacteria.

### Nsp13 and DDX5, a Trick to Enhance Viral RNA Unwinding?

**Figure 11:**
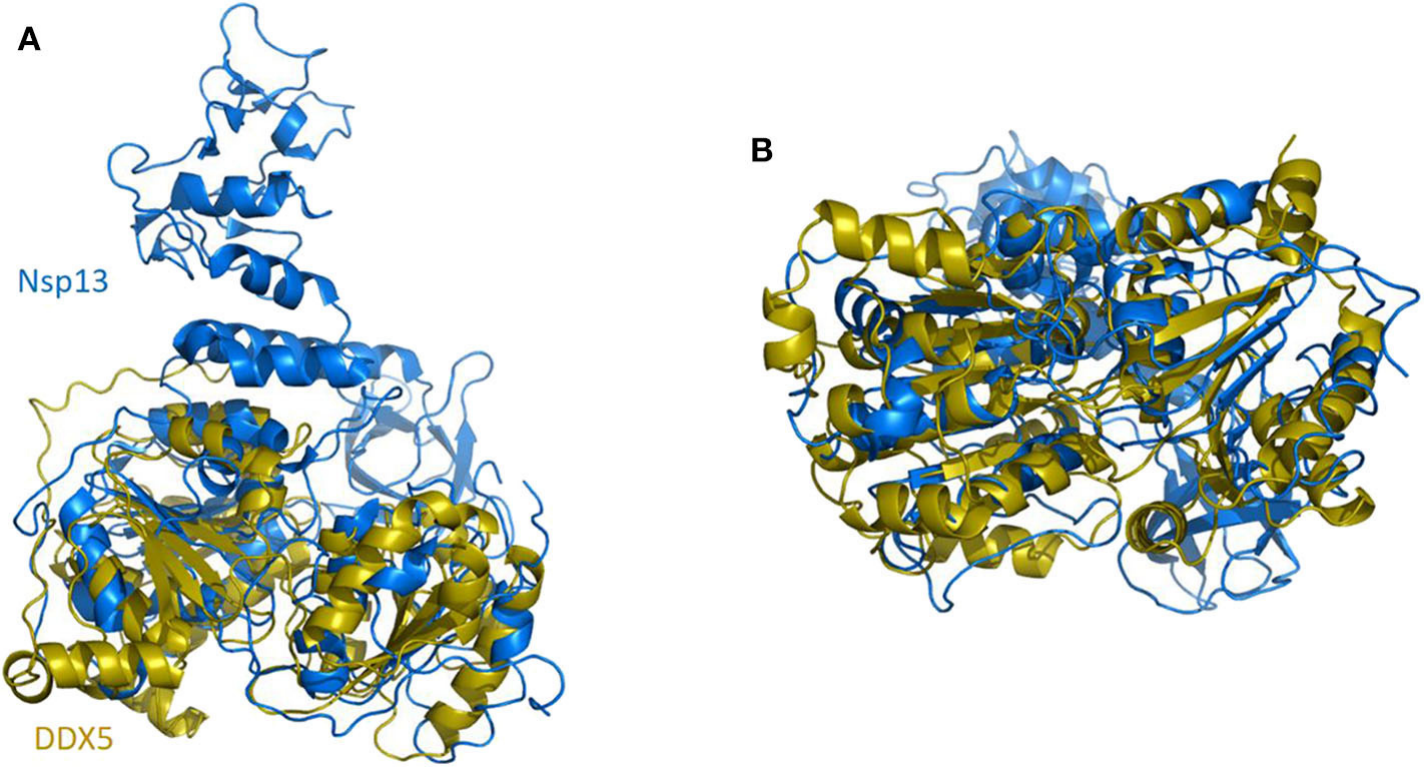
Superposition of SARS-CoV-2 Nsp13 on DDX5 homology model. Cartoon representation of DALI superposed Nsp13 (blue) and the D1D2 core of DDX5 homology model (gold). **(A)** Side and **(B)** bottom view.

In SARS-CoV replication, the helicase Nsp13 interacts with DDX5 (Chen J. Y. et al., 2009). Interestingly, inhibition of DDX5 by RNA interference results in the suppression of viral replication (Chen J. Y. et al., 2009). This finding indicates a pro-viral function of DDX5 in coronavirus infection through Nsp13 binding, suggesting that the host helicase may act as a coactivator to enhance viral genome transcription and virus proliferation. A search in the DALI database shows that Nsp13 structurally resembles several homologous human DDX helicases, as judged by the high values of Z scores and low root mean square deviation (rmsd) between backbone atoms (Table 2). This structural similarity occurs despite the low sequence identity between Nsp13 and these helicases (ranging between 8 and 12%, Table 2), which include DDX5 (Table 2, Figure 11).

As previously described, crystallographic and solution structural studies of DDX3X have proven that two DDX molecules function cooperatively to unwind dsRNA, in a mechanism that has been proposed as a general unwinding mechanism of DDX helicases (Song and Ji, 2019), and therefore applying to DDX5. Although which regions of Nsp13 and DDX5 are involved in binding is unknown, it is tempting to speculate that Nsp13 may form a pre-unwound dimer with DDX5, as observed for DDX3X and proposed for all DDX helicases (Song and Ji, 2019). Consistent with this hypothesis, recent studies have reported thatNsp13 of SARS-CoV has a strong affinity for duplex RNA as a substrate for unwinding, in a reaction which requires high ATP concentrations to unwind duplex RNA (Jang et al., 2020). By crosslinking dsRNA in conjunction with DDX5, Nsp13 would be able to efficiently unwind viral RNA even in the early stages of infection, when the concentration of Nsp13 is still low, by exploiting its interaction with DDX5.

### Sequestration of DDX5 as Possible Viral Evasion Mechanism From Inflammatory Response

Recently, DDX5 has been shown to interact with the diacylglycerol kinase ζ (DGKζ), an activator of the NF-κB transcription factor, an essential innate immunity/inflammation modulator. Knockdown of DDX5 only repressed NF-κB transcriptional activity, thus attenuating this essential branch of the innate immune response (Tanaka et al., 2020). Thus, similarly to the case of DDX3X and DDX1 reported above, binding of Nsp13 to DDX5 may play a dual role: enhancing viral RNA transcription and repressing innate immunity.

## Conclusions

While human coronaviruses usually cause mild symptoms, three highly pathogenic coronaviruses have emerged in the last years, causing serious diseases: SARS-CoV in 2002, MERS-CoV in 2012, and SARS-CoV-2 in 2019, responsible for the current COVID-19 pandemic. A detailed understanding of the SARS-CoV-2 life cycle and its interactions with the host cell proteome is mandatory to develop effective therapeutic strategies and is currently being actively pursued. However, while such studies are being carried out for SARS-CoV-2, precious information can also be derived from previous studies on the other coronaviruses. In fact, both structural and functional studies have identified host cell proteins as molecular players of coronaviral hijacking mechanisms and described ways exploited by the virus to replicate more efficiently by taking advantage of its host.

As discussed in this review, various DDX helicases, including DDX3X, DDX5, and DDX1, are known to play important roles in the replication cycle of coronaviruses. The viral proteins responsible for DDX-mediated hijacking mechanisms are highly conserved among coronaviruses, an observation that suggests common pathways used by these viruses to exploit host proteins for their own advantage. Here, based on available structural data integrated with homology modeling, we explore possible interactions between human DDX and coronavirus proteins. It appears that the explored mechanisms work to modulate different aspects of viral life. In many instances, sequestration of DDX helicases can have a dual pro-viral role: enhancing key steps of the virus life cycle such as RNA replication/transcription, and, at the same time, repressing the innate immune response at various levels. Based on the structural similarity of the involved viral proteins among different coronaviruses, we thus hypothesize that similar mechanisms are operating also for SARS-CoV-2. Thus, we suggest that DDX helicases could represent useful novel targets for antiviral therapy also against SARS-CoV-2, as already validated for other RNA viruses (Brai et al., 2016, 2019, 2020b). Indeed, proteomic studies have identified interactions between SARS-CoV-2 proteins and various DDX helicases. However, work needs to be done to understand the roles that these host proteins play in the viral life cycle. Since DDX helicases are not essential for cell viability, gene silencing/disruption approaches could be used to measure the infection cycle of SARS-CoV-2 in cells ablated for specific DDX proteins. In addition, biophysical studies could be used to map the interaction domains between DDX and the cognate viral proteins. Also, for those viral proteins endowed with enzymatic activities, such as Nsp13 and Nsp14, biochemical studies could reveal the effects of DDX interactions on their individual catalytic activities.

## Author Contributions

RB, FS, and AR: conceptualization. RB, MR, and AR: methodology and software. MR, FS, and AR: formal analysis. FS and RB: data curation. RB: original draft preparation, supervision, and funding acquisition. RB, FS, and GM: writing review and editing. All authors contributed to the article and approved the submitted version.

## Conflict of Interest

The authors declare that the research was conducted in the absence of any commercial or financial relationships that could be construed as a potential conflict of interest.
