# Supplementary material for: Host DDX Helicases as Possible SARS-CoV-2 Proviral Factors: A Structural Overview of Their Hijacking Through Multiple Viral Proteins
Source: Front Chem. 2020 Dec 10;8:602162. doi: 10.3389/fchem.2020.602162 (PMC7769135; doi:10.3389/fchem.2020.602162)
Supplement: Supplementary file 1 [file Table_1.docx]

**Host DDX helicases as possible SARS-CoV-2 proviral factors: a structural overview of their hijacking through multiple viral proteins**

**Supplementary Material**

**Table S1.** Sequence identities of SARS-CoV-2 proteins mentioned in this manuscript with their homologs. The binary alignments were performed using Lalign Server tool.

|  | **Sequence identity (%)** | | | |
| --- | --- | --- | --- | --- |
| **SARS-CoV-2** | SARS-CoV | MERS-CoV | IBV-CoV | MHV |
| N protein | 90.5 | 71 | 27.5 | 33.1 |
| Nsp10 | 97.1 | 59.3 | 54.8 | 52.5 |
| Nsp12 | 96.4 | 71.1 | 61.9 | 66.3 |
| Nsp13 | 99.8 | 71.4 | 58 | 67.3 |
| Nsp14 | 95.1 | 62.6 | 52 | 82.5 |
